# Heritage and Stigma. Co-producing and communicating the histories of mental health and learning disability

**DOI:** 10.1136/medhum-2016-011083

**Published:** 2017-05-30

**Authors:** Rob Ellis

**Keywords:** History, Mental health care

## Abstract

University engagement with mental health services has traditionally been informed by the vocational and pedagogical links between the two sectors. However, a growth in the interest in public history and in the history of mental healthcare has offered new opportunities for those in the humanities to engage new audiences and to challenge perceptions about care in the past. The introduction of the ‘impact agenda’ and related funding streams has further encouraged academics to contribute to historical debates, and to those concerning current services. One such example of this is the Arts and Humanities Research Council funded Heritage and Stigma project at the University of Huddersfield, which was conceived to support mental health and learning disability charities in the exploration and dissemination of their own histories. Using this project as a case study, this paper will draw on primary source material to reflect on the opportunities and challenges of working in partnership with such groups. In particular, it will consider the need to address issues of stigma and exclusion in tandem with a critical understanding of the moves to ‘community care’ instigated by landmark legislation in the form of the 1959 Mental Health Act. Overall, it provides evidence of an inclusive, coproductive model of design and highlights the positive contribution to communicating mental health made by those based in the humanities.

In 2012, the Arts and Humanities Research Council (AHRC) launched its ‘Research for Community Heritage’ initiative in collaboration with the Heritage Lottery Fund (HLF).^1^ Its aim was to encourage engagement, communication and collaboration between community groups and academic researchers based in Institutes of Higher Education (HE). One aspect of the call was the expectation that community partners would play a central role in the co-design and co-production of research. For those involved, the benefits of this approach were clear. Academics could form creative partnerships and working relationships beyond the confines of their own disciplines and, crucially, access a new strand of funding. Similarly, groups beyond HE could also access funds and call on academic expertise to help shape community projects.

As AHRC literature has pointed out—‘academic research has a natural affinity with a community's wish to discover its own heritage’,^2^ but behind this apparently benign and innovative call was a complex web of issues. In particular, the context of funding grew out of sceptical views which challenged the value of arts and humanities research. Writing from a US perspective, Gary Olson has described this scepticism as a ‘crisis’ and points to a more recent and concerted challenge from influential policy makers.^3^ In the UK, there is evidence of a politicisation of some funding streams with the AHRC's delivery plan in 2010 stating that its Connected Community programme would contribute to the government's initiatives on localism and the ‘Big Society’.^4^ More generally, the Research Excellence Framework (REF) included units of assessment whereby universities had to demonstrate ‘an effect on, change or benefit to the economy, society, culture, public policy or services, health, the environment, or quality of life beyond academia’.^4^ This new ‘Impact’ subcategory represented 20% of the overall REF assessment in 2014. Here, impact case studies were judged in terms of both ‘reach’ and ‘significance’, with outcomes determining a significant part of university funding in the long term.^5^ This is not to say that the role of impact was unwelcome across the humanities. Some academics saw this as an opportunity to communicate their work to new audiences and this is reflected in the sheer weight of projects supported by the AHRC.^6^

Despite the lure of funding and the chance to broaden the appeal of research, there are philosophical reasons why the chance to work with community groups is appealing. The focus of this short article is on history and, for some, the appeal of partnership working is linked to the challenges to the perceived ownership of and authority over the past that arose with the history workshop model pioneered by Raphael Samuel.^7^ Samuel questioned ‘the assumption that knowledge filters downwards’, and the *History Workshop Journal* has been publishing the work of academically trained historians that gives serious attention to grass-roots projects since its first issue in 1976.^8^ In these cases collaborative working is not new and already questions are being asked about an impact agenda that bases the value of research on the assumption that knowledge is communicated only in one direction — from academic(s) to a community or other group.^9^ For those with an interest in communicating mental health this is significant. While some argue that ‘…the study of history can have real benefits for key mental health stakeholders in the here and now’, finding a connection between historical understanding and current practice can be a challenge.^10^ Moreover, there are many reasons why historians do not want to be involved with current stakeholders. This might be about the focus of their research, but equally there is the detrimental effect that ‘impact’ activities can have on an individual's workload, their time for research and, as a corollary, their prospects for promotion.^7^ Nevertheless, it is clear that ‘impact’ is not going away—at least not in the short term. As one Dean has predicted, by 2020 we will ‘have impact coming out of our ears’.^6^

With these things in mind, this article will offer some initial reflections on the opportunities offered by new funding streams for historians and others to work together to communicate both mental health and learning disability. To do so, it will draw on the experience of the ‘Heritage and Stigma’ project which was based at the University of Huddersfield and funded by the AHRC's Connected Communities programme. Heritage and Stigma was designed to support community interest in histories and the first section of this article will explain its origins in more detail. It will then move on to explore the common ground between historians and healthcare practitioners and then, crucially, the areas in which history can prove to be relevant to current practice. The final section explores how history, specifically, and the humanities more generally, can be used to break down barriers—whether this is between academics and the ‘real-world’ or, as in this case, between those with learning disability or mental health diagnoses and others. Given the limitations of space, it is important to state at the outset that this article focuses on an academic viewpoint, rather than a reflection on the multitude of voices in the process. These, it is hoped will be published as subsequent reflections where they can be given the space and time needed to explore the issues more fully. In the meantime, a theme of this article is the contribution that researchers in the arts and humanities can make in the co-production and co-design of ‘real world’ outputs but also what they can learn from the process. While the origins of co-production can be found in citizen participation in the development of public services in the USA in the 1970s, many in the humanities are only just starting to consider its value in the production of new research and new knowledge.^11–13^ Some of this is intimately connected with the policy changes described above and this article aims to add something to that discussion.

## Background to Heritage and Stigma

The development of Heritage and Stigma grew out of previous public engagement but its funding was dependent on ‘community’ interest in the academic histories of mental health and learning disability. In this case, the community was represented by Leeds Mencap, a learning disability charity, and St Anne's Community Services, an organisation that covers a large part of the north of England. While St Anne's also supports people with learning disability, its focus for this project was on mental health. Both organisations were interested in exploring their histories and they wanted to include their clients and service users within the process. Significantly, these were separate projects, each with their own funding from the HLF, and operating to different timescales but they both approached the author with a view to him joining their project teams. Subsequent funding from the AHRC allowed two academic members of staff time and resources to support these initiatives allowing them to become members of the project steering groups, offering advice and support in the development and delivery of key project themes. This included a broader understanding of the past and help with archival research and oral history interviews, as well as project managing and mentoring student volunteers.

Of course, these projects are not the first ones that involved academics working in partnership on similar kinds of issues. The Open University's interdisciplinary Social History of Learning Disability Research Group, for example, was formed in 1994. Its members recognise that ‘people with learning disabilities are expert in their own lives, and have historical knowledge, viewpoints and skills to contribute’.^14^ However, two things made these projects different. First, AHRC and HLF funding actively encouraged community groups to seek out academic partners and vice versa. Indeed, before any bids for funding were made, initial, separate discussions took place as part on an existing AHRC funded project titled *Sound, Craft, Vision, Place*. This funding allowed the University to facilitate a number of events whereby representatives of community groups could find out more about the HLF's *All Our Stories* funding—and, significantly, explore the potential to work in partnership.^15^ Both Leeds Mencap and St Anne's applied separately for, and were awarded, relatively small amounts of HLF funding*.* This was then followed by a bid by the author of this article to the AHRC's *Connected Communities* fund to enable each of these groups to work in partnership with the University. Second, while the discrete nature of the Leeds Mencap and St Anne's bids to the HLF meant that, while there were some differences in each of their projects, the outcomes in each case were broadly the same. Leeds Mencap wanted to create materials in time for its 60th anniversary which would be showcased in an exhibition at Leeds City Museum. By contrast, St Anne's had no significant anniversary in mind, but they too looked to present a museum exhibition, which was hosted at the Tolson Museum in Huddersfield. These were opportunities to communicate the histories of mental health and learning disability in new ways but the involvement of museums meant further negotiation about how to display this appropriately. In each case, the project teams were dependent on museum professionals to offer advice and support on the opportunities and limitations offered by their chosen dissemination methods. Thus while an end point of academic research could influence some of the content, it could not hope to shape it all. As a result, the co-design and co-production of outputs, and the success of the project, was dependent on finding areas of commonality for all concerned.

## Historians, current practice and some common ground

While some historians of mental health and learning disabilities have personal stories to tell, for most, their studies remain an academic exercise rooted in the past.^16^ Either way, there are some parallels when it comes to exploring ‘histories from below’. Reflecting on the shifting nature of the historiography of psychiatry and its invasion by new social historians in the 1970s and 1980s, Andrew Scull has drawn attention to the influences of history from below on his own work and that of others.^17^ Highlighting a clear connection to his own, pivotal *Museums of Madness* (1979), Scull has singled out Michael MacDonald's *Mystical Bedlam* (1981) and Roy Porter's *Mind Forg'd Manacles* (1987) as key examples of new explorations of the ‘poor and powerless’.^17^ Indeed, in Porter's article, subtitled ‘Doing Medical History from Below’, he called on historians to lower their gaze so that they might better understand patient-doctor roles in the past.^18^ Although a rallying call to medical historians, his work on Georgian madness can be seen as a more specific extension of this. Getting ‘inside the heads of the ‘mad’’, he argued, was central to an understanding of treatments in the past that did not focus solely on relationships with, and the power of, medical professionals.^19–21^

For some, patient testimonies have offered the potential to communicate the histories of mental health in a new way and to throw the connection between institutions and society into ‘heightened and revealing relief.’^22^ For others, the focus on history from below meant exactly that. Michael Ignatieff's review of five publications that included *Museums of Madness,* encouraged authors to explore the agency of the various actors in the committal process and question the assumption that the ‘state was the hammer, and the working class only and always the anvil’.^23^ Since that time, the attempt to understand and reflect on patient perspectives has been an important feature of many historical studies. For others, the patient story, while important, was not necessarily defined on class grounds alone. Gender is clearly important here, but so too have been the differences between individual patients. As has been pointed out elsewhere, histories of madness that include mad ‘eminences’ rather than truly lowly and anonymous madmen and madwomen are not necessarily histories from below.^24^ To a degree, such comments reflect the availability of source materials and while things like diaries and letters can help to illuminate individual case studies, they are often difficult to come by. As a result, the use of archives that contain documents with a medical and/or an administrative focus mean that the unpicking of patient narratives is not straightforward. For those with research interests in the 20th century and beyond, the availability of and access to multiple narratives is potentially wider, and although not everyone chooses to go down that route, there are some good examples of academic studies which focus on patient perspectives.^25^ ^26^

Writing as an academic historian with no experience of current practice, this will to place patients at the heart of historical analysis appears to reflect initiatives that place service users and clients at the heart of decision-making processes. Indeed, although service user advocacy has its own history, their involvement in policy-oriented research and the evaluation of services had been going on since the late 20th century.^27^ ^28^ While it may be stretching the point to draw a direct connection, the awareness of the importance and centrality of the voice and views of people at the sharp end of procedure and policies seems to provide some common ground, and so it proved with the Heritage and Stigma project. Beyond, this, however, the success of the project was dependent on how the histories of care were relevant to its aims, and this meant challenging some commonly held views about care in the past.

## Institutions and community care and their links to the project

To be clear, the focus of both these projects was on the two charities and the work they did in supporting their clients in the community. With hindsight it is easy to see the end of what Andrew Scull termed ‘asylumdom’ as shorthand for its dehumanising failure to cure or adequately care for the people held in large institutions. The Mental Health Act of 1959 has been described a ‘landmark’, in changing attitudes and in forcing the pace of that change but it is clear that public attitudes did not change overnight.^29^ Similarly, while Kathleen Jones has described 1959 as a legislative revolution that followed important clinical and administrative revolutions in the 1950s, others have since challenged the view that this was a key turning point.^30^ Central to this have been the motivations for change, with questions being asked about the supposed efficacy of new pharmacology in the 1950s and the savings to be made from the closure of large public institutions.^31^ ^32^ More importantly, it is clear that the ‘ideal’ of a shift towards community care took time to become a reality. In the 1970s, the publication of government white papers, entitled *Better Services for the Mentally Handicapped* (1971) and *Better Services for the Mentally Ill* (1975), reiterated a commitment to community care. At the same time they recognised that the shift away from the dominant model of institutional care had occurred in a piecemeal way.^33^ It was only in the 1980s that the closure programme accelerated and, again, the economic arguments for their closure have proved to be important.^34^ While limitations of space do not permit an in-depth discussion of this, there remains a sense that the Whiggish or progressive view of closure remains a compelling one. Significantly, in terms of communicating the story of mental health and learning disability, it is this academic contribution to an understanding of the past that is important in making the project relevant.

First and foremost, it would be easy to fall into the trap of presenting a triumphalist history of present day care. This is something that numerous commentators have been inclined to do over the last 200 years-or-so, but for many in the sector now care cannot always be explained in terms of its superiority. Furthermore, part of the rationale for building specialist institutions was to protect individuals from the hurtful comments of others. John Conolly's description of the ‘sport and mockery’ afforded to ‘harmless maniacs’ wandering at large before the 19th century provided useful ammunition in the professional or Whiggish histories.^35^ Conolly was the resident physician at the Middlesex County Asylum at Hanwell and his role in the introduction of the ‘non-restraint’ of patients helped to emphasise the apparent positives of care in enlightened asylums.^36^ As a corollary, depictions of the apparent failure of extrainstitutional care prevailed for a long time. A rare piece of evidence from a patient who had been in an asylum in the 19th century, offers an individual and personal example of that ‘mockery’. After leaving the asylum and returning home, he found that he was ‘laughed to scorn by inferior men than myself, [and] my brother insults me since I came out of the asylum’. He concluded that ‘the people are worse out of the asylum than in….’^37^ There is some sense here that the mockery of some individuals was linked to the stay in the asylum—that institutions stigmatised people—but for those who worked in that sector the emphasis was on the safety and security of institutional care. John Langdon Down, Medical Superintendent of the Royal Earlswood Asylum, wrote of the ‘feeble minded children’ whose lives were made ‘wretched by teasing’.^38^ In the 21st century, the shift from medical to social models of disability make such views seem hopelessly outdated, but such fears played a role when institutions began to close. In 1995, a spokesman for the Parents and Relatives Association of Meanwood Park Hospital wrote,

Half of it is now boarded up and its occupants, apart from “managers,” are being relocated to an increasingly dangerous community. By all means encourage those who can cope, to return to decent homes in the community. But for others a “hospital village” is the ideal place for them to be looked after. It also offers respite care for others at a reasonable cost to the taxpayer.^39^

Significant here is the use of the term ‘hospital village’, as Meanwood had opened as a colony following the passing of the Mental Deficiency Act in 1913. The history of Leeds City Council's decision to build the colony is still waiting to be written, but the letter cited above reminds us that its story was not simply one of segregation and seclusion.

Of course, for lots of people, the reality of institutionalisation was not as positive as the care that is presented in these sources. Huge numbers of families and individuals sought to resist it and the existence of mental health survivor groups seek to shed light on the darker side of ‘care’. Moreover, despite attempts of administrators and staff to engage local communities with hospitals, the perception of them as isolated and secluded was and is pervasive.^40^ These elements help to explain why the closure of these places has been presented in progressive or Whiggish terms, but this does not mean that all of the issues, such as mockery, isolation and the need for respite care in some cases have simply disappeared.^34^ ^41^ Moreover, we need to be aware that, the organisations themselves were not separate from the changing landscape of care in the 20th century. While it might be convenient to present the shift from large-scale institutional care as a ‘break’ or a ‘revolution’, the reality is those more complex links between organisations and the societies they served. Take, for example, the advertisement in *The Times* in 1957*,* for the National Society for Mentally Handicapped Children, the organisation that would be renamed as Mencap. Alongside an image of a young boy, the text read

A DARK SHADOW HANGS OVER THIS CHILD this Christmas—and over 150 000 others like him. Please give in thankfulness that you have been spared the tragedy of a mentally handicapped child.^42^

This appeal for funds would allow the Society to give advice, support and help to distressed parents and children, and would sponsor research into the causes and prevention of mental handicap. The focus on the tragedy of the mentally handicapped child contrasts sharply with the aims of the organisation now. A shift in representations came in 1992 with a relaunch that sought to ‘revolutionise and update the society's image’.^43^ While the moves from the term mental handicap to learning disability took a little while longer, the ‘little Stephen’ image was dropped and, instead of focusing on the negative or the tragic, new materials included ‘making the most of life’.^44^

**Figure 1:**
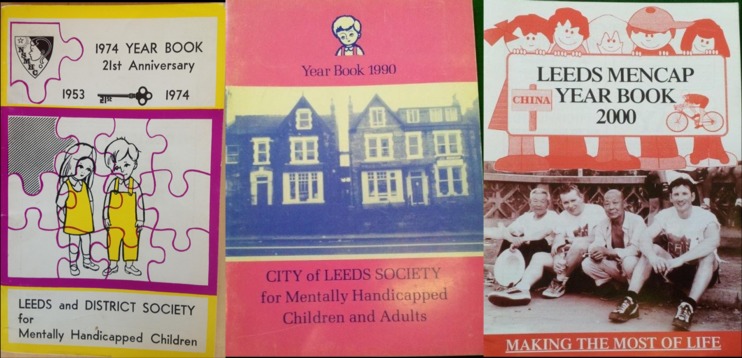
Examples of Leeds Mencap year books, 1974, 1990 and 2000.

Cleary, these images reflect what can only be described as positive changes, but they highlight the fact that the closure of large-scale institutions is just one part of the story. More important, are the realities of individual perspectives. Indeed, in a discussion of the factors affecting Scope's name change from the Spastics Society, one member of the team commented that changing peoples' attitudes to disability was less straightforward than an organisational rebranding.^45^ These examples emphasise the fact that attitudes within organisations have changed too and they became even more relevant when, in the course of the Leeds Mencap project, a series of yearbooks were found that reflected those changes on a local level (figure 1).

**Figure 2:**
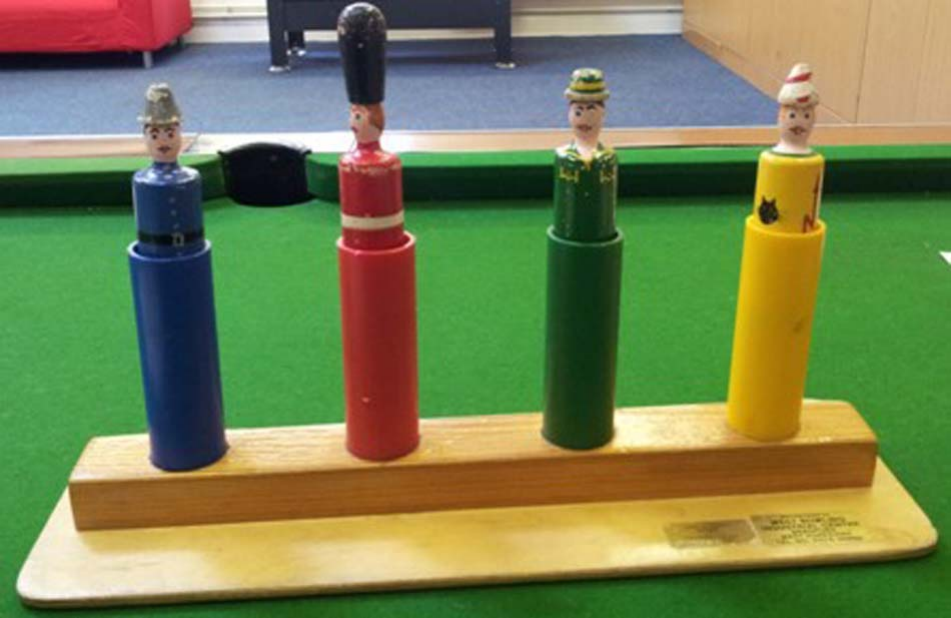
A toy from Leeds Mencap.

These images demonstrate a changing perspective from within Mencap, but the closure programme that stretched into the 1980 s and 1990 s was important for other reasons. A good example of this was a staff member who was able to talk about the toy that had been used by her in the Meanwood Park Hospital and was still used by her at Leeds Mencap today (figure 2). The toy was a simple but colourful depiction of four figures which individually bounced up and down on a spring when pressed. Although it was still used in the nursery at Leeds Mencap, it had been commissioned as a bespoke piece of equipment in a period before the development of specialist manufacturers. Such a toy had the potential to be overlooked in the story of the closure of the hospital and, without an understanding of the changes that had taken place, may have appeared incongruous. There is of course, the potential for more research here but it offered a physical and individual connection between the age of institutions and the age of care in the community. More importantly, it again reflected the multiple narratives that make up the histories of mental health and learning disabilities.

Without this historical focus it is unlikely that these things would have been uncovered or contextualised within the project. Feeding this in to steering group meetings and mentoring those involved in the co-production of project outputs meant that the wider academic understanding of the past was, and is important in avoiding contradictions that automatically assume that the past was ‘bad’ and the present is good. In these cases we can see a deeper narrative of the moves away from institutionalisation that includes changes within the organisations themselves.^46^ ^47^ Moreover, while these things are important, historically speaking, they are also important to understanding the challenges of mental ill-health and learning disabilities in the 21st century. Concerns about the standards of care now, or the relatively parlous state of public spending in both mental health and learning disability services are regular features of news reports in the UK. Similarly, despite the shifts in the dominant narratives of care, a regular feature of campaigns in these areas seeks to address the issues of stigma and the apparent intolerance of people with little knowledge or understanding of their impact on peoples' lives. In this respect, presenting the moves from institutionalisation to community care as a panacea, however tempting, is flawed.

## Communicating histories: some thoughts on co-production and the present

Here then, there are indeed links between academic research and a ‘community's wish to discover its own heritage’.^2^ As has been pointed out elsewhere, however, ‘we should not assume [academic research] must be the one and only thing that makes a change’ and, instead, it must be seen in terms of how it can be used to create mutually beneficial relationships.^7^ The process of communication has to work both ways, and others too, have reached similar conclusions. Work in the development of museum exhibitions in relation to mental health, for example, has sought to challenge the notion of the exhibition as the end point of a topic by working in collaboration with people with lived experience.^48^ Again, the theme here is patients, service users and clients and those who are often excluded from historical accounts.^48^

For historians and others, there are calls for further research into some of the more recent changes to care and to place service users at the heart of that story.^i^ The obvious advantage is that it allows for opportunities to shift the gaze away from medical or administrative records, but any projects that seek to do this are not necessarily collaborative. By contrast, this project included joint meetings with staff and service users to discuss the shape and direction of the project. Crucially, co-designing and co-producing outputs allowed individuals the opportunity to represent themselves in their own terms—not just as patients or service users—but as individuals. Similarly, there are opportunities not just to think about triumphal declarations about the superiority of paradigms of care but to highlight ongoing issues and concerns.

**Figure 3:**
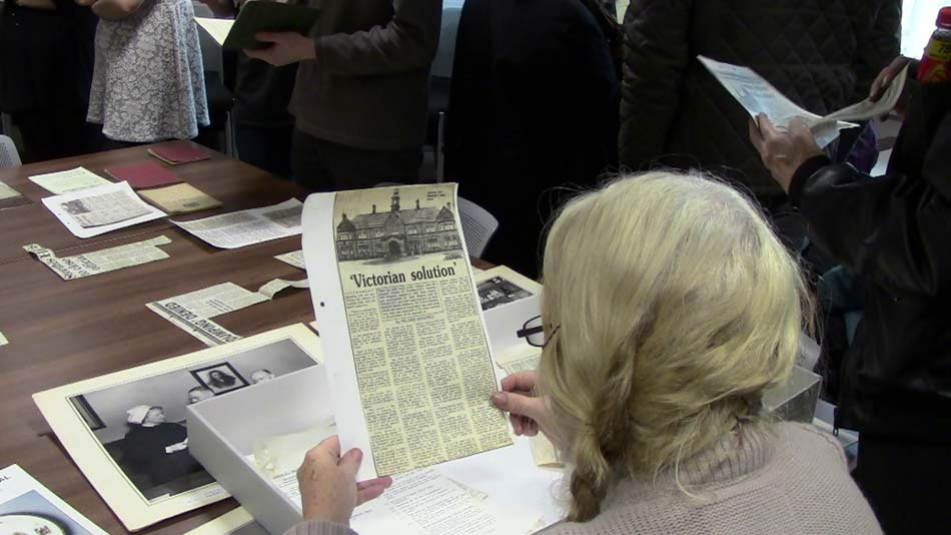
Groups from St Anne's team, identifying areas of research.

Historical sources, such as those described above, and an understanding of their strength and limitations can be used to demonstrate the ‘ordinariness’ of mental illness and learning disability now and in the past; they can evidence the challenges and the positive and negative responses to them. All of these help us tell a longer-term story and even highlight some of those issues such as acceptance and belonging that remain the same in some cases—even if the dominant paradigm of care has changed. On its own, however, this does not seem to be enough. Reflecting on the two projects, there has to be some recognition that not everyone is interested in or inspired by history as a subject, or stories from the past. Nevertheless, history had and has a contribution to make—in ensuring that the links to the past were logical, and in providing an opportunity to break down barriers. The mixed project teams meant that service users helped design and shape the project, rather simply serving as vehicles for research. Projects like this allow community groups into an otherwise off-limits campus and break down the barriers between universities and the wider world, and in this case those who live with mental ill-health or learning disability and those who do not. Figure 3 offers an example of a team visit to the University archive but group trips and meetings took place on and off campus at various sites including Museums and other archives. In each case, these groups comprised service users and clients as well as Mencap and St Anne's staff, and staff and students of the University. In these examples, it is clear that the models of co-production and the construction of mixed teams were as important as the outputs, but it was also about sharing of knowledge and it was the funding that allowed the space and resources to facilitate these discussions.

Had this been a purely academic research exercise, I may have chosen other areas to focus on but it was only by working in partnership that the teams uncovered previously unseen and unheard materials that could form the basis of a discrete academic research project. It is unlikely that without the funding these sources and these opportunities would have arisen. On a very basic level then the uncovering of source material was a positive but ‘embracing the entanglement’^13^ of the projects had other advantages. My involvement enabled me to learn more about the everyday realties of mental health and learning disability from people with lived experience. In turn, this has allowed me to consider more fully the human aspects involved in what are often dry medical and administrative archival records. More prosaically, rather than being an end point of research, working in this way has allowed me to gain a greater understanding of the past, which will feed into both future research projects and teaching at the University.

## Conclusions

Although there are clear parallels in their bids to communicate mental health, and in this case learning disability, the concrete connections between historical endeavour and current practice are not always immediately obvious. Some of this is about the realities of the day job, but equally an agreement on a clear rationale for working together can be an early stumbling block. What these initial thoughts on the process of co-producing outputs have emphasised is the common ground between historians, practitioners and services users and the things we can learn from each other. An understanding of the history of the post-1959 world was important, but on its own it was not enough to draw project partners in. While there may be other pathways to impact for historians of mental health and learning disability, and other ways to communicate the findings of their academic research, working with current stakeholders seems to offer the best and most logical opportunities. Again, most, if not all historians know this already—but in doing so, there are things that academics can learn from the process, as well as the knowledge they can bring to it. In this case, the success of the project depended on the other partners connected with it and co-producing work that meant something to everyone concerned. In this respect the outputs, although well received, seemed less important than the process of making them. For Leeds Mencap and St Anne's this was a way to break down barriers. Staff at both organisations spoke of the positive impact that the project had on some of their clients and, in turn the process helped me reconsider the ways I viewed the past.

Having said all that, this way of working brings with it its own challenges, not least the amount of time that was spent on the administration and organisation by each of the project partners. This was something we all seemed to underestimate and this has probably something to do with the fact that these were some of our first steps in co-producing outputs. Similarly, there was the ‘newness’ of impact as a measurable outcome to consider and its place within the day job. While these things can be taken into account and are something to be considered in any future bids, there has to be some reflection on where projects like this fit within the workload of academics that includes teaching, research and administration. Impact adds a fourth element and while it takes time it does not necessarily reduce the need for academic outputs. Of course, some subjects lend themselves to impact more readily than others, and some academics are drawn to it for other reasons, but having a starting point that includes existing research makes staff involvement in projects such as this one potentially difficult—more so for early career researchers. Examples such as the one above demonstrate the impact of humanities and the impact of impact. However, if funders, policy makers and universities are serious about the impact that the humanities can have on wider society, they have to find the space for it and they have to reward the effort that goes into it accordingly.

## Footnotes

i The shift to community care had been described as ‘reinstitutionalistation or transinstitutionalisation’ as the decrease in conventional psychiatric beds has been met with a corresponding increase in beds in forensic psychiatry, places in supported housing, involuntary hospital admissions and people in prison. The paper concludes with calls for ‘specific research’ in a number of areas—not least the experience of patients in these different forms of institutions.^27^
